# RYBP expression is associated with better survival of patients with hepatocellular carcinoma (HCC) and responsiveness to chemotherapy of HCC cells *in vitro* and *in vivo*

**DOI:** 10.18632/oncotarget.2598

**Published:** 2014-10-18

**Authors:** Wei Wang, Jianwen Cheng, Jiang-Jiang Qin, Sukesh Voruganti, Subhasree Nag, Jia Fan, Qiang Gao, Ruiwen Zhang

**Affiliations:** ^1^ Department of Pharmaceutical Sciences, School of pharmacy, Texas Tech University Health Sciences Center, Amarillo, TX, USA; ^2^ Cancer Biology Center, School of Pharmacy, Texas Tech University Health Sciences Center, Amarillo, TX, USA; ^3^ Liver Cancer Institute, Zhongshan Hospital, and Key Laboratory of Carcinogenesis and Cancer Invasion, Ministry of Education, Fudan University, Shanghai, China; ^4^ Institute of Biomedical Sciences, Fudan University, Shanghai, China

**Keywords:** RYBP, Prognosis, Combination therapy, Apoptosis, HCC

## Abstract

RYBP is a member of the polycomb group (PcG) proteins that typically act as transcriptional repressors *via* epigenetic modification of chromatin. The present study was designed to investigate the role of RYBP in HCC progression, chemosensitivity, and patient survival, and to explore the underlying molecular mechanism(s). In this study we investigated the expression of RYBP in 400 pairs of human HCC tissues and matched noncancerous samples. The effects of RYBP on HCC tumor growth and metastasis and chemosensitivity were determined both *in vitro* and *in vivo*. We herein demonstrate that the RYBP expression in HCC tissue samples was signiﬁcantly lower than that in matched noncancerous liver tissues. Clinically, the low expression of RYBP was an independent predictor of a poor prognosis in patients with HCC. In *in vitro* HCC models, enforced RYBP expression inhibited cell growth and invasion, induced apoptosis, and increased the chemosensitivity of the cells, while RYBP knockdown led to the opposite effects. Furthermore, RYBP expression was induced by cisplatin, and adenovirus-mediated RYBP expression inhibited HCC tumor growth and sensitized HCC to conventional chemotherapy *in vivo*. Our results demonstrate that reactivating RYBP in cancer cells may provide an effective and safe therapeutic approach to HCC therapy.

## INTRODUCTION

Hepatocellular carcinoma (HCC) is one of the most common malignancies, and approximately 30,640 new cases are diagnosed annually in the United States [1], with a trend toward an increasing incidence and prevalence. HCC is associated with a poor prognosis and limited therapeutic options [2]. Surgical resection of the tumor may yield a better prognosis for patients with resectable disease. However, the current systemic chemotherapy has produced unsatisfactory results, and several cytotoxic agents, such as cisplatin, doxorubicin and 5-florouracil (5-FU), have resulted in little or limited benefits [2]. A better understanding of the biological processes of hepatocarcinogenesis has created a great opportunity to identify molecular targets for more effective therapeutic intervention. Recent studies have indicated that a loss of tumor suppressor function and amplification/mutation of oncogenes have critical roles in the development and progression of HCC. Dysfunctions of several signaling pathways regulating apoptosis in hepatocytes are unique in the molecular pathogenesis of HCC, providing novel molecular targets for treating HCC [3].

Polycomb group (PcG) proteins are transcriptional repressors that epigenetically modify chromatin and participate in the establishment and maintenance of cell fates [4]. These proteins are crucial for many biological processes, including self-renewal and differentiation, and cancer [5,6]. RYBP (RING 1 and YY1-binding protein) is a newly identified member of the PcG proteins. RYBP, which belongs to the non-canonical polycomb repressive complex 1 (PRC1), functions as a transcriptional repressor in mammalian cells by interacting with and repressing the transcriptional activity of several sequence-specific transcription factors, such as YY1 (Yin Yang 1), GABPB1 (GA-binding protein subunit beta-1) and E2F6 (E2F transcription factor 6) [7,8].

Intriguingly, RYBP also has transcription repression-independent (non PcG) functions [9-16]. There have been reports showing that RYBP preferentially inhibits the proliferation of malignant cells, but not non-transformed cells, *in vitro* [9,11]. RYBP interacts with FADD (Fas-associated protein with death domain), caspase-8 and caspase-10 through their death effector domains (DED), enhancing the formation of the death-inducing signaling complex (DISC) and promoting Fas-mediated apoptosis [12]. Additionally, RYBP has been suggested to act as a negative regulator of cell invasion [13]. RYBP has also been suggested to be a target of miRNA-27 and 29, which affect physiological processes such as skeletal myosis [14,15]. Our recent study has identified RYBP as a novel regulator of the oncogene MDM2 [16]. Mechanistically, RYBP stabilizes and activates p53 by interacting with MDM2 and decreasing the MDM2-mediated p53 degradation [16]. It also induces p53-dependent G1 phase arrest and is involved in the p53 response to DNA damage [16]. In our initial study with patient primary tumor tissue samples, we found that the RYBP level is reduced in human lung and liver cancer tissues compared to the corresponding normal tissues [16]. However, the potential role of RYBP in HCC is largely unknown.

In light of the previously published reports and our preliminary findings, we hypothesized that RYBP can be exploited as a novel target for human HCC therapy. In the present study, for the first time, we systemically investigated the levels of RYBP expression and the linkage between RYBP deregulation and survivals of patients with HCC. Using *in vitro* and *in vivo* HCC models, we determined the role of RYBP in cancer cell response to chemotherapy. We first found that RYBP was downregulated in human HCC cell lines and tumor specimens and that RYBP was an independent predictor of survival in patients with HCC. We further demonstrated that RYBP inhibited HCC cell growth through induction of apoptosis *in vitro* and *in vivo*. We also determined the effects of chemotherapeutic agents on the RYBP expression, and the role of RYBP in the chemosensitivity *in vitro* and *in vivo*. The results of these studies provide initial evidences supporting that the restoration of RYBP expression may be a new approach to targeted therapy for HCC and that RYBP may be a useful biomarker for predicting the prognosis of patients with HCC.

## RESULTS

### RYBP is downregulated in HCC tissues, and low expression of RYBP correlates with a poor prognosis in HCC patients

**Fig.1:**
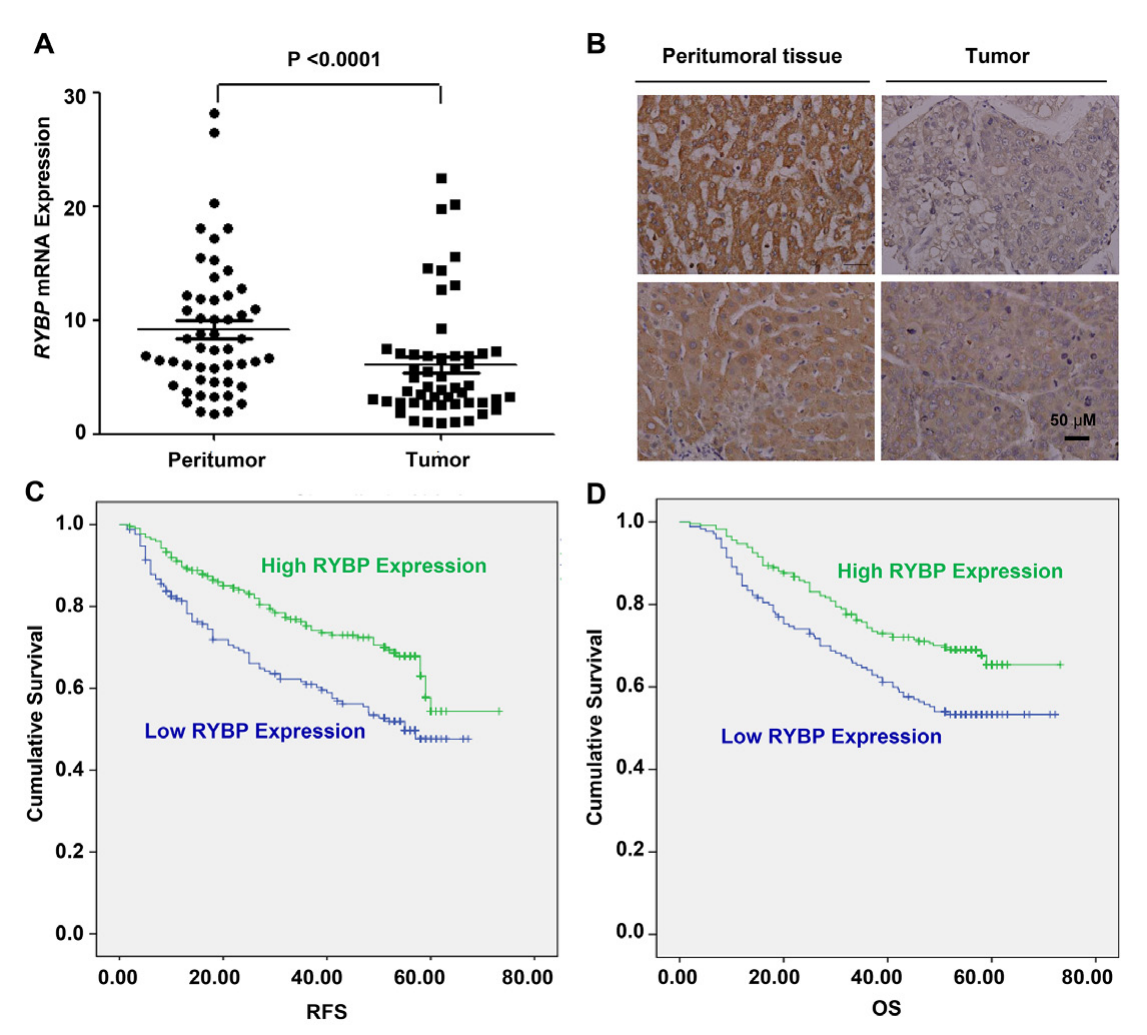
RYBP is frequently downregulated in HCC tissues, and a low level of RYBP is associated with a poor prognosis (A) The quantitative real-time analyses of the RYBP mRNA levels in HCC and non-malignant adjacent tissue samples (n=52). (B) The immunohistochemical analysis of the RYBP expression in HCC tissues compared with paired non-malignant peritumoral tissues. (C) The recurrence-free survival (RFS) rates of 400 patients with HCCs were compared between the RYBP-low or -high groups using the Kaplan–Meier method (log-rank test). (D) The overall survival (OS) rates of the 400 patients with HCCs were compared between the RYBP-low or -high groups using the Kaplan–Meier method (log-rank test).

To determine whether there was an association between the RYBP expression in HCCs and the disease outcome, we first examined the relative RYBP mRNA levels in 52 pairs of human HCC tissue samples by quantitative real-time PCR. Our results showed that the transcriptional level of RYBP was significantly downregulated in HCC specimens compared to corresponding adjacent normal liver tissue samples (P < 0.0001) (Fig. 1A). Tissue microarrays (TMAs) from 400 patients with HCCs were also examined by immunostaining (Fig. 1B shows the staining in representative samples). We found that 378 (94.5%) HCC cases had positive tumor cell RYBP expression, with varied intensities. There were 152 (37.0%) cases with weak RYBP intensity in the tumors cells, 167 (40.6%) with moderate intensity, and 59 (14.4%) with strong intensity staining for RYBP. In contrast, the peritumoral liver cell RYBP expression was strong in most cases (92.2%), with the other 7.8% of cases having moderate intensity staining for RYBP.

To evaluate the associations between the RYBP level and the tumor biology, we compared the clinicopathological features with the RYBP expression. The patients with low RYBP expression were significantly more likely to exhibit aggressive clinicopathological features. For example, the RYBP-low patients harbored more tumors with poor differentiation (p = 0.001) and had increased serum γGT levels (p = 0.022) (Table 1).

**Table 1:** The correlations between the clinicopathological findings and tumor RYBP expression

Characteristic	RYBP		
Low	High	P value
Age, years			
≤ 53	84	112	0.799
> 53	90	114	
Gender			
Male	143	191	0.534
Female	31	35	
Hepatitis history			
Yes	153	203	0.681
No	19	22	
AFP (ng/ml)			
≤ 20	67	83	0.715
> 20	107	143	
γGT (U/l)			
≤ 54	80	130	0.022
> 54	94	96	
Liver cirrhosis			
Yes	31	31	0.261
No	143	195	
Tumor size (cm)			
≤ 5	101	142	0.331
> 5	73	84	
Tumor encapsulation			
None	95	125	0.887
Complete	79	101	
Tumor multiplicity			
Single	151	189	0.381
Multiple	23	37	
Tumor differentiation			
I-II	112	178	0.001
III-IV	62	48	
Vascular invasion			
Yes	63	66	0.137
No	111	160	
TNM stage			
I	99	138	0.173
II	62	63	
III	13	25	
BCLC stage			
A	60	91	0.299
B	51	69	
C	63	66	

NOTE. Pearson's chi-square test was used to determine the significance.

Abbreviations: AFP, alpha-fetoprotein; γGT, gamma-glutamyl transferase; TNM, tumor-node-metastasis; BCLC, barcelona-clinic liver cancer.

There was an excellent correlation between the RYBP intensity and the RFS (recurrence-free survival) or OS (overall survival). The median follow-up period was 52.1 months (range, 2.0–73.2; SD, 18.0). The RFS and OS (in brackets) rates at one, three and five years post-hepatectomy were 87% (92%), 70% (71%) and 54% (60%), respectively, for the whole study population. As expected, RYBP-low expression (scores 0 and 1) patients had a significantly poorer RFS and OS than did the patients with RYBP-high expression (scores 2 and 3) (Figs. 1C and 1D). The five-year RFS and OS rates were 61% and 66% for the RYBP-high patients, compared with 46% and 52% for the RYBP-low patients, respectively. In a multivariate analysis, the tumor RYBP status was defined as an independent prognostic factor for both the RFS and OS. RYBP-low patients were nearly two times more likely to suffer from relapse than RYBP-high patients (HR = 0.62, 95% CI = 0.45-0.85) (Tables 2 and 3). Thus, these results indicate that RYBP expression is a valuable predictor of survival in patients with HCC.

**Table 2:** The results of the univariate and multivariate analyses of the prognosis factors associated with the recurrence-free survival in patients with HCC

Variable	Univariate		Multivariate	
	HR (95% CI)	P value	HR (95% CI)	P value
Age, years (≤ 53 vs. > 53)	1.08 (0.78-1.49)	0.648		NA
Gender (female vs. male)	1.67 (1.02-2.73)	0.042		NA
Hepatitis history (no vs. yes)	1.35 (0.75-2.43)	0.325		NA
AFP (ng/ml) (≤ 20 vs. > 20)	1.04 (0.75-1.45)	0.811		NA
γGT (U/l) (≤ 54 vs. > 54)	2.36 (1.69-3.28)	0.000	2.16 (1.55-3.03)	<0.001
Liver cirrhosis (no vs. yes)	2.44 (1.35-4.41)	0.003	2.91 (1.61-5.28)	<0.001
Tumor differentiation (well vs. poor)	1.11 (0.85-1.45)	0.464		NA
Tumor size (cm) (≤ 5 vs. > 5)	1.41 (1.02-1.94)	0.040		NA
Tumor multiplicity (single vs. multiple)	1.48 (0.96-2.28)	0.075		NA
Tumor encapsulation (complete vs. none)	1.09 (0.79-1.50)	0.596		NA
Vascular invasion (no vs. yes)	1.57 (1.13-2.18)	0.008		NA
TNM stage (I vs. II vs. III)	1.42 (1.13-1.79)	0.003		NA
BCLC stage (A vs. B vs. C)	1.41 (1.16-1.70)	<0.001	1.33 (1.10-1.61)	0.004
RYBP (high vs. low)	0.58 (0.42-0.80)	0.001	0.62 (0.45-0.85)	0.003

NOTE. A Cox proportional hazards regression model was used for the analyses.

Abbreviations: AFP, alpha-fetoprotein; γGT, gamma-glutamyl transferase; HR, hazard ratio; CI, confidence interval; TNM, tumor-node-metastasis; BCLC, barcelona-clinic liver cancer; NA, not applicable.

**Table 3:** The results of the univariate and multivariate analyses of the prognosis factors associated with the overall survival in patients with HCC

Variable	Univariate		Multivariate	
	HR (95% CI)	P value	HR (95% CI)	P value
Age, years (≤ 53 vs. > 53)	1.05 (0.76-1.44)	0.776		NA
Gender (female vs. male)	1.24 (0.79-1.96)	0.344		NA
Hepatitis history (no vs. yes)	1.18 (0.67-2.08)	0.571		NA
AFP (ng/ml) (≤ 20 vs. > 20)	1.41 (1.00-1.98)	0.051		NA
γGT (U/l) (≤ 54 vs. > 54)	2.09 (1.50-2.92)	<0.001	1.69 (1.21-2.37)	0.002
Liver cirrhosis (no vs. yes)	1.63 (0.97-2.74)	0.065		NA
Tumor differentiation (well vs. poor)	1.63 (1.16-2.28)	0.005	1.49 (1.06-2.10)	0.023
Tumor size (cm) (≤ 5 vs. > 5)	2.25 (1.63-3.11)	<0.001	1.58 (1.11-2.23)	0.011
Tumor multiplicity (single vs. multiple)	2.01 (1.36-2.93)	<0.001	1.71 (1.15-2.54)	0.008
Tumor encapsulation (complete vs. none)	1.20 (0.87-1.65)	0.273		NA
Vascular invasion (no vs. yes)	1.90 (1.39-2.65)	<0.001		NA
TNM stage (I vs. II vs. III)	1.77 (1.42-2.21)	<0.001		NA
BCLC stage (A vs. B vs. C)	1.68 (1.38-2.04)	<0.001	1.34 (1.07-1.68)	0.012
RYBP (high vs. low)	0.60 (0.44-0.83)	0.002	0.66 (0.47-0.91)	0.012

NOTE. A Cox proportional hazards regression model was used for the analyses.

Abbreviations: AFP, alpha-fetoprotein; γGT, gamma-glutamyl transferase; HR, hazard ratio; CI, confidence interval; TNM, tumor-node-metastasis; BCLC, barcelona-clinic liver cancer; NA, not applicable.

### RYBP overexpression and knockdown affect the growth, apoptosis, and invasion of HCC cells

We also examined the RYBP expression in immortalized human normal hepatocytes (CL48) and hepatoma-derived cell lines. RYBP was expressed in all hepatoma cell lines. In most of the hepatoma cell lines, both the mRNA and protein levels of RYBP were lower than those in the normal hepatocytes (Supplementary Figs. 1A and 1B).

**Fig.2:**
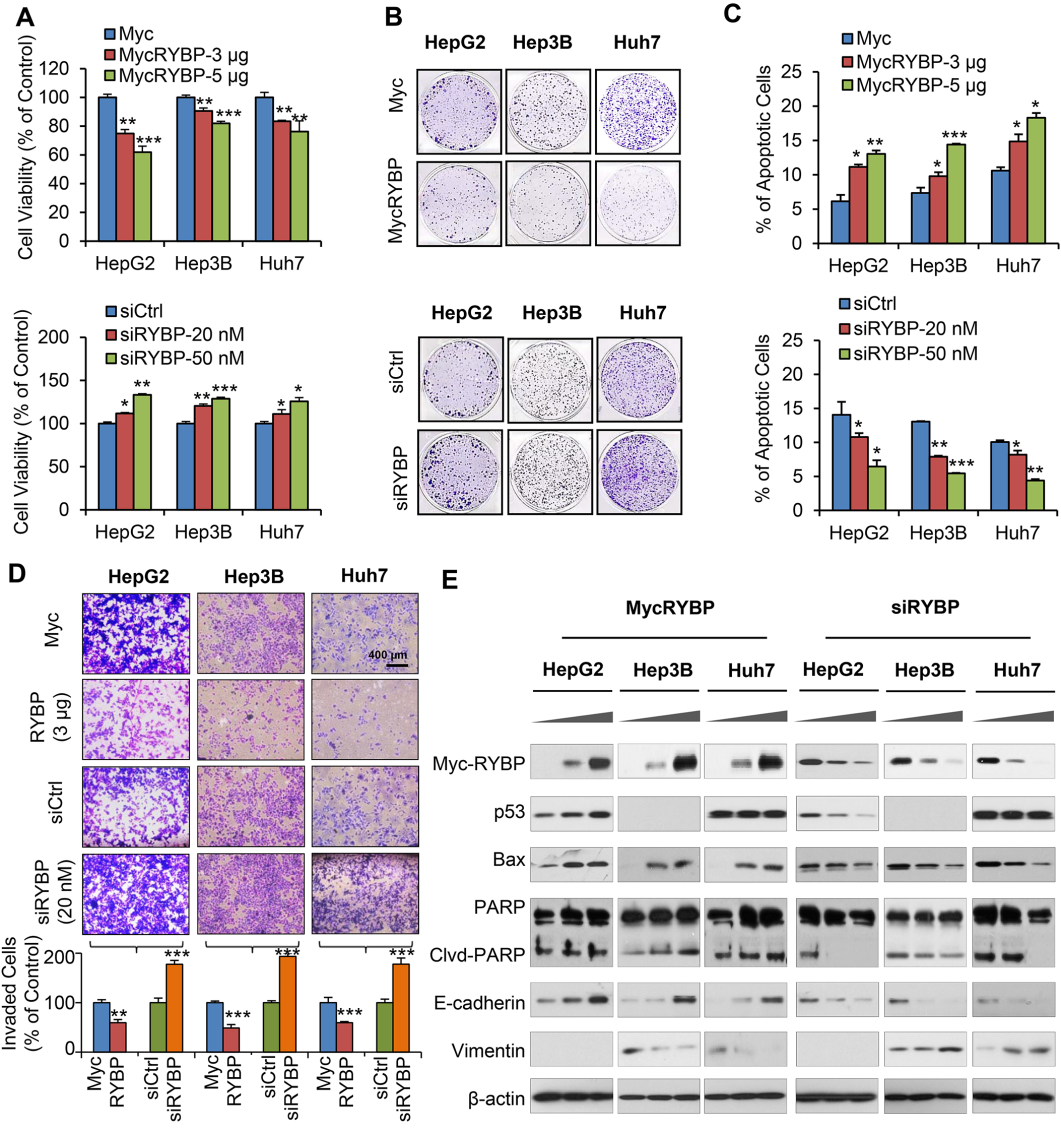
RYBP overexpression and knockdown affect the growth, apoptosis, and invasion of HCC cells HepG2, Hep3B and Huh7 cells were transiently transfected with an RYBP plasmid or empty vector for 24 h or with RYBP siRNA or a non-targeting control siRNA for 36 h. The cell viability was determined by the MTT assay (A); the cell survival was determined by the colony formation assay (B); cell apoptosis was determined by the Annexin V-FITC method (C); cell invasion was determined by the Transwell invasion assay (D); and the expression of RYBP and apoptosis- and metastasis-related proteins was determined by immunoblotting assays (E). All assays were performed in triplicate (^*^P<0.05, **P<0.01, ***P<0.001).

Given the clinical significance of RYBP in HCC, we wanted to know whether manipulating the RYBP expression could lead to biological effects in HCC cells. Our recent study shows that RYBP is a positive regulator of p53 [16], but the possible p53-independent function of RYBP may also be explored, which would provide a complete picture for RYBP's role in carcinogenesis and cancer development and progression. Based on different genetic backgrounds (p53 status and protein expression levels of RYBP), three cell lines were chosen in this study: HepG2 (p53 wild-type; RYBP middle), Hep3B (p53 null; RYBP low), and Huh7 (p53 mutant; RYBP high). First, HCC cells were transiently transfected with the Myc-RYBP plasmid or siRYBP, and the cell growth was determined by using the MTT and colony formation assays. As shown in Fig. 2, enforced expression of RYBP inhibited the cell viability (Fig. 2A, upper panel) and colony formation (Fig. 2B, upper panel) of HCC cells in a dose-dependent manner, regardless of the p53 status. In contrast, knockdown (KD) of endogenous RYBP improved the HCC cell viability (Fig. 2A, lower panel) and colony formation (Fig. 2B, lower panel), independent of p53.

To investigate how RYBP suppresses HCC cell growth, we investigated whether RYBP impacts apoptosis in HCC cells. As shown in Fig. 2C, transient overexpression (OE) of RYBP induced dose-dependent cell apoptosis (Fig. 2C, upper panel). In contrast, RYBP KD cells exhibited low levels of apoptosis in all three HCC cell lines (Fig. 2C, lower panel). Moreover, we also examined whether RYBP has a role in HCC metastasis. The transwell assays showed that RYBP OE substantially inhibited the invasive activity of the cells, whereas RYBP KD significantly promoted the invasive potential of HCC cells (Fig. 2D). The levels of treatment (3 μg MycRYBP; 20 nM siRYBP, and 300 MOI AdRYBP) and treatment time (24 h) used in the invasion assay did not cause significant inhibition of cell growth or apoptosis, so the effects of the treatment on invasion activity were unlikely to be linked to cell survival.

### RYBP overexpression and knockdown affect the expression of apoptosis- and metastasis-related proteins in HCC cells

To elucidate the underlying mechanism(s) responsible for the anti-HCC activity of RYBP, we examined the effects of RYBP OE and KD on the expression of apoptosis- and metastasis-related proteins in HCC cell lines. As shown in Fig. 2E, in agreement with the increase in apoptosis, we observed that RYBP OE increased the expression level of Bax and led to increased PARP cleavage. The p53 levels in the treated HepG2 cells were elevated, but the mutant p53 levels in Huh7 cells were unchanged. We next analyzed the effects of RYBP OE on several markers of the epithelial-to-mesenchymal transition (EMT). RYBP OE markedly increased the level of E-cadherin and decreased the level of vimentin in a dose-dependent manner in all three HCC cell lines. However, RYBP KD affected the expression of these proteins in the opposite way. These findings demonstrated that RYBP suppresses the malignant phenotype of HCC cells at least partly by modulating the levels of apoptosis- and metastasis-related proteins through both p53-dependent and -independent mechanisms.

### Adenoviral RYBP overexpression suppresses the malignant properties of HCC cells *in vitro*

**Fig.3:**
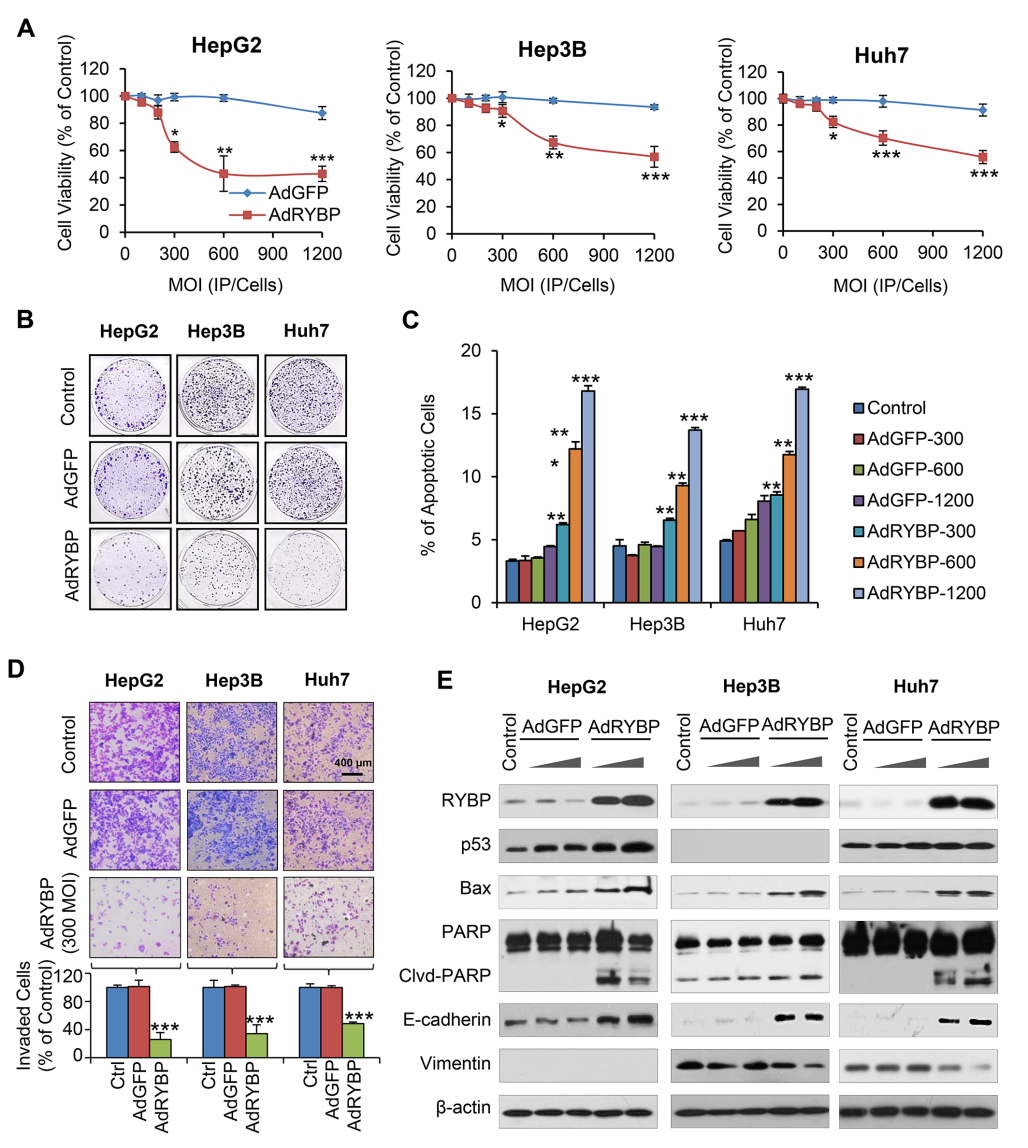
AdRYBP infection suppresses the malignant properties of HCC cells HepG2, Hep3B and Huh7 cells were infected with AdRYBP or AdGFP for 24 h, then the cell viability was determined by the MTT assay (A); the cell survival was determined by the colony formation assay (B); apoptosis was determined by the Annexin V-FITC method (C); cell invasion was determined by the Transwell invasion assay (D); and the expression of RYBP and apoptosis- and metastasis-related proteins was determined by immunoblotting assays (E). All assays were performed in triplicate (*P<0.05, **P<0.01, ***P<0.001).

To further validate whether virus-mediated delivery of RYBP can lead to anti-tumor effects, we generated a replication-deficient adenovirus driving the expression of RYBP (AdRYBP), and infected the HCC cells. In agreement with the results obtained using plasmid transfection, AdRYBP infection decreased the viability of HCC cells in a concentration-dependent manner (Fig. 3A); at a MOI of 1200 infectious particles per cell (IP/cell), AdRYBP, but not AdGFP, inhibited the cell viability by about 57% (P < 0.001), 43% (P < 0.001), and 44% (P < 0.001) in HepG2, Hep3B, and Huh7 cells, respectively. The inhibition of cell colony formation was also observed in HCC cells that were infected with AdRYBP, but not those infected with AdGFP (Fig. 3B).

AdRYBP infection also induced apoptosis in all three HCC cell lines, regardless of the p53 status (Fig. 3C). In the HepG2, Hep3B, and Huh7 cells, treatment with AdRYBP at a MOI of 1200 IP/cells increased the apoptotic index by 5.1- (P < 0.001), 3.0- (P < 0.001), and 3.5-fold (P < 0.001), respectively. However, no significant apoptosis occurred in HCC cells infected with AdGFP up to 1200 IP/cells. Moreover, the suppression of cell invasion (Fig. 3D) was also observed with the AdRYBP treatment. In addition, the expression levels of proteins known to be involved in the apoptosis and invasion in HCC cells were modulated in cells with AdRYBP infection, compared with the controls (Fig. 3E).

### RYBP overexpression and knockdown affect the HCC cells' response to chemotherapeutic agents *in vitro*

**Fig.4:**
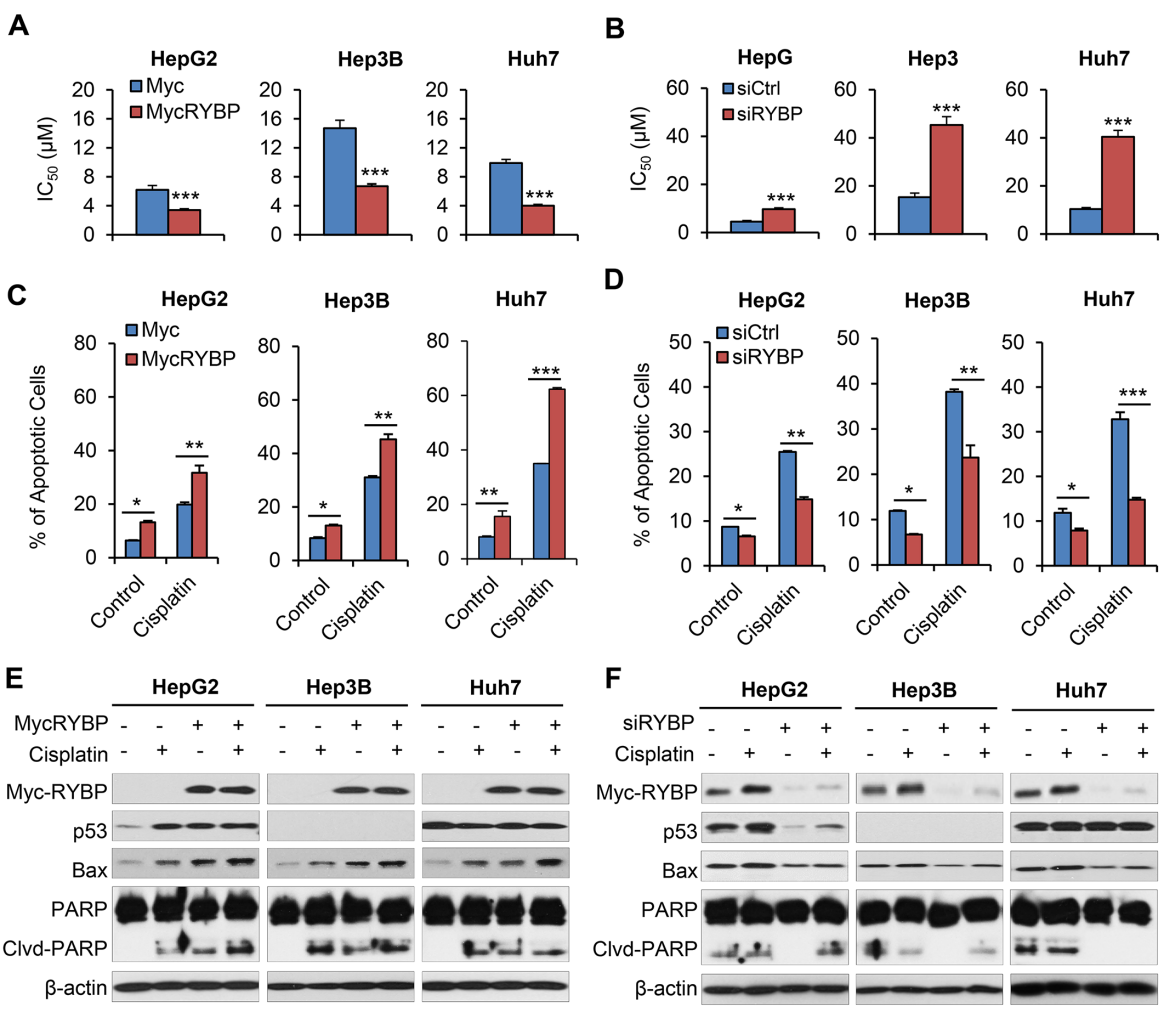
RYBP overexpression and knockdown affect the chemosensitivity of HCC cells to cisplatin HepG2, Hep3B and Huh7 cells were treated with cisplatin and an RYBP plasmid (24 h) or RYBP siRNA (48 h), then the IC_50_ values were determined by the MTT assay (A and B); apoptosis was determined by the Annexin V-FITC method (C and D); and the expression of RYBP and apoptosis- and metastasis-related proteins was determined by immunoblotting assays (E and F). All assays were performed in triplicate (*P<0.05, **P<0.01, ***P<0.001).

Combing gene therapy and chemotherapy can represent a successful approach for cancer treatment [17]. To determine if RYBP affects the chemosensitivity of HCC cells, we first tested whether RYBP OE would sensitize HCC cells to treatment with cisplatin or 5-FU, which have been the most effective agents used as systemic chemotherapy for HCC [18]. As shown in Fig. 4, RYBP OE improved the response to cisplatin, reducing the IC_50_ values in HepG2 (6.2 ± 0.6 vs 3.4 ± 0.2 μM), Hep3B (14.7 ± 1.1 vs 6.7 ± 0.3 μM), and Huh7 cells (9.9 ± 0.5 vs 4.0 ± 0.2 μM) (all P < 0.01; Fig. 4A). In contrast, RYBP KD increased the IC_50_ values to cisplatin by 2.2-, 3.0-, and 3.9-fold in HepG2, Hep3B, and Huh7 cells, respectively (all P < 0.001; Fig. 4B). Similar results were observed in RYBP OE or KD HCC cells treated with 5-FU (all P < 0.001; Supplementary Figs. 2A, B).

We further examined the effects of RYBP on the cisplatin- and 5-FU-induced apoptosis in HCC cells. As shown in Fig. 4C, 10 μM cisplatin alone increased the apoptotic index by about 3.1-, 3.7-, and 4.3-fold in HepG2, Hep3B, and Huh7 cells, respectively, compared to vehicle-treated cells. However, RYBP OE dramatically increased the apoptotic index following cisplatin treatment by 5.0-fold, 5.4-fold, and 7.7-fold, respectively, compared to the control. In contrast, RYBP KD cells treated with cisplatin caused 1.7-fold, 2.0-fold, and 1.2-fold decreased apoptotic index, compared to the control, respectively (Fig. 4D). Similar results were observed in RYBP OE or KD HCC cells when they were treated with 5-FU (Supplementary Figs. 2C,).

We also examined the expression of apoptosis-related proteins in RYBP OE and KD HCC cells that were treated with or without cisplatin. As shown in Fig. 4E, cisplatin treatment induced RYBP expression. RYBP OE enhanced the cisplatin-induced increase in p53 in HepG2 cells, and improved the cisplatin-mediated upregulation of Bax and PARP cleavage. The RYBP KD reversed these effects of cisplatin in HCC cells (Fig. 4F).

### RYBP overexpression by adenoviral infection sensitizes HCC cells to chemotherapy *in vitro*

**Fig.5:**
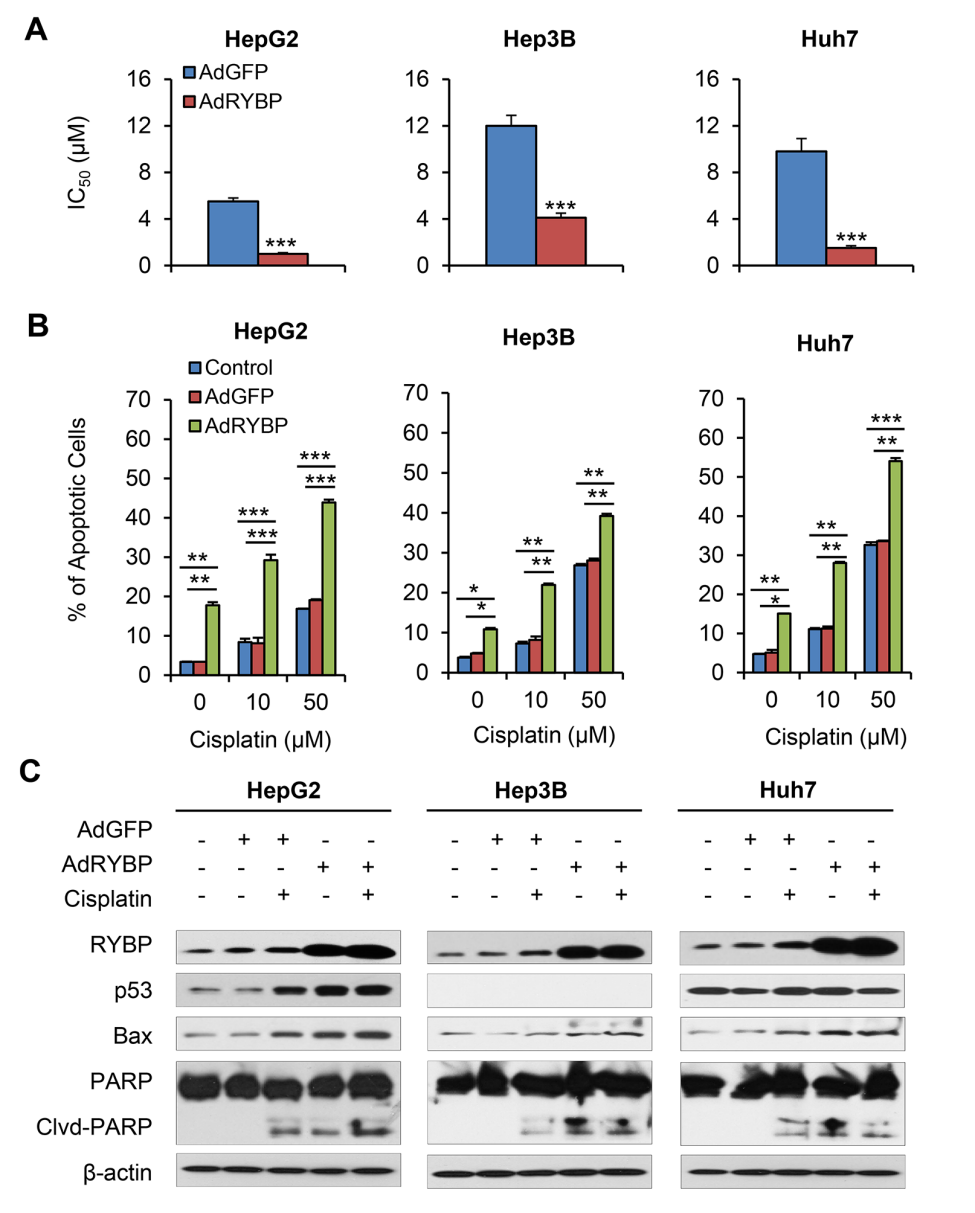
AdRYBP infection increases the chemosensitivity of HCC cells to cisplatin HepG2, Hep3B and Huh7 cells were treated with cisplatin and an AdRYBP plasmid (24 h), then the IC_50_ values for cisplatin were determined by the MTT assay (A); cell apoptosis was determined by the Annexin V-FITC method (B); and the expression of RYBP and apoptosis- and metastasis-related proteins was determined by immunoblotting assays (C). All assays were performed in triplicate (*P<0.05, **P<0.01, ***P<0.001).

Similarly, combining AdRYBP with these conventional therapies dramatically decreased the IC_50_ values (all P < 0.001; Fig. 5A and Supplementary Fig. 3A). The combination therapy also resulted in significant increases in the percentage of apoptotic cells compared to cisplatin or 5-FU treatment alone (all P < 0.01; Fig. 5B and Supplementary Fig. 3B). The Western blotting analyses indicated that the expression changes of several apoptosis- and chemoresistance-related proteins by AdRYBP infection could be responsible for the RYBP-induced chemosensitization to cisplatin in HCC cells (Fig. 5C).

### RYBP inhibits the growth of HCC and sensitizes HCC tumors to chemotherapy *in vivo*

**Fig.6:**
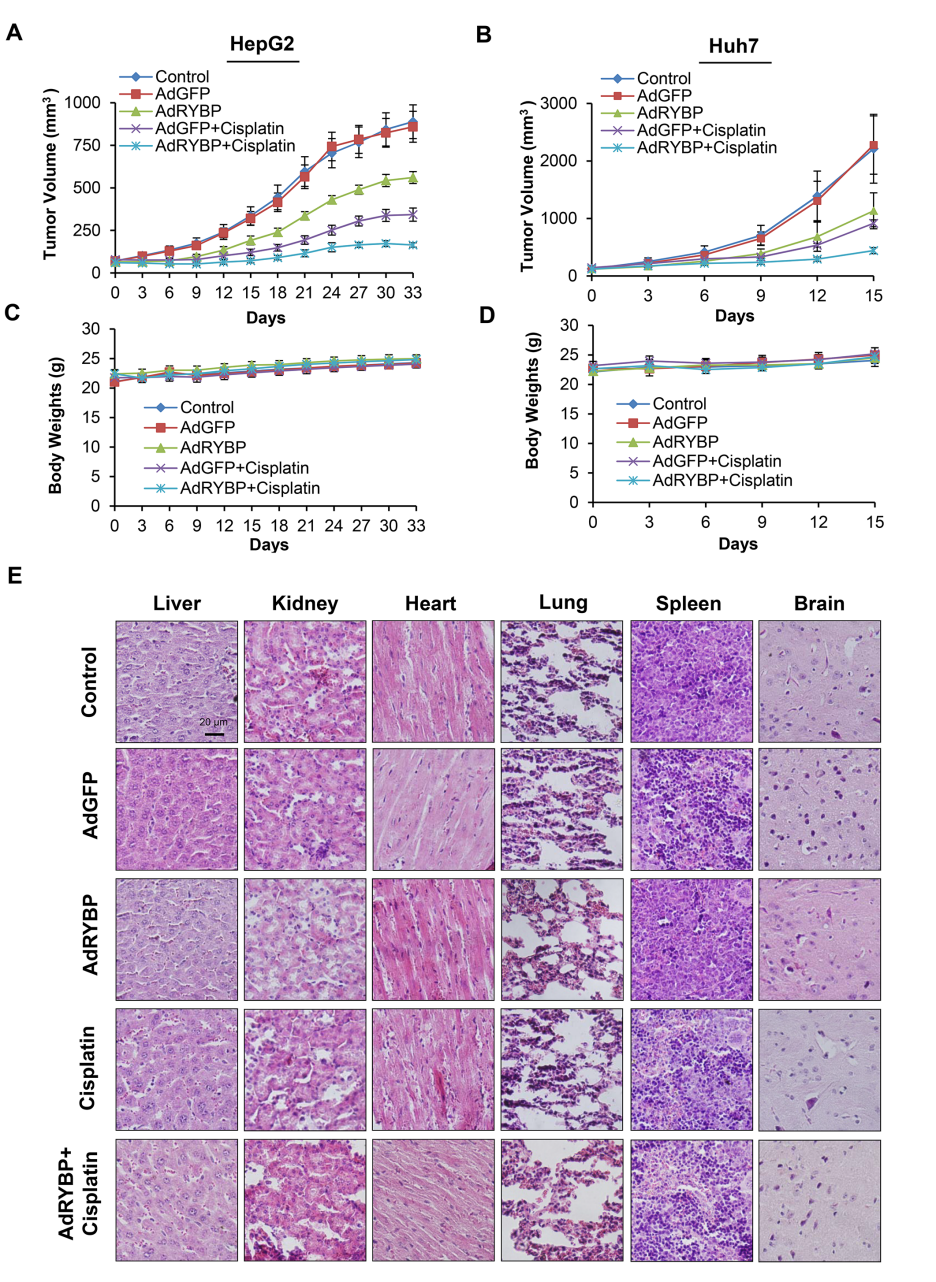
RYBP inhibits the growth of HCC and sensitizes HCC tumors to chemotherapy *in vivo* A total of 2×10^9^ infectious particles (IP) of AdGFP or AdRYBP were administered by intratumoral injection every three days to nude mice bearing HepG2 (A) or Huh7 (B) tumors, and cisplatin was administered to the mice at 5 mg/kg by intraperitoneal injection once per week. The animals were monitored for changes in body weight as a surrogate marker for toxicity in both the HepG2 (C) and Huh7 (D) xenograft models. (E). At the end of the experiment, H&E staining of the paraffin sections of other tissues (heart, lung, liver, kidney, spleen and brain) from mice bearing HepG2 xenograft tumors were performed. All images represented the series sections.

To validate the anti-HCC effects of RYBP *in vivo*, nude mice bearing HepG2 and Huh7 xenograft tumors were treated by intratumoral injection of AdRYBP. Our results showed that the AdRYBP treatment inhibited the growth of HepG2 and Huh7 xenograft tumors by about 37% (P < 0.001) (Fig. 6A) and 49% (P < 0.001) (Fig. 6B), respectively, whereas AdGFP treatment did not affect the tumor growth compared with untreated mice (Figs. 6A and 6B). Cisplatin alone decreased the tumor growth by 62% and 59%, respectively. However, the combination of cisplatin with AdRYBP significantly reduced tumor growth by 81% and 80%, respectively (Figs. 6A and 6B). Of note, there were no remarkable changes in the average body weights in both animal models (Figs. 6C and 6D) and no differences in histology examinations among all the treatment and control groups in all the normal tissues (heart, lung, liver, kidney, spleen and brain) in the mice bearing the HepG2 xenografts (Fig. 6E), indicating that AdRYBP infection was safe at the effective therapeutic doses when used alone or in combination with chemotherapeutic agents.

**Fig.7:**
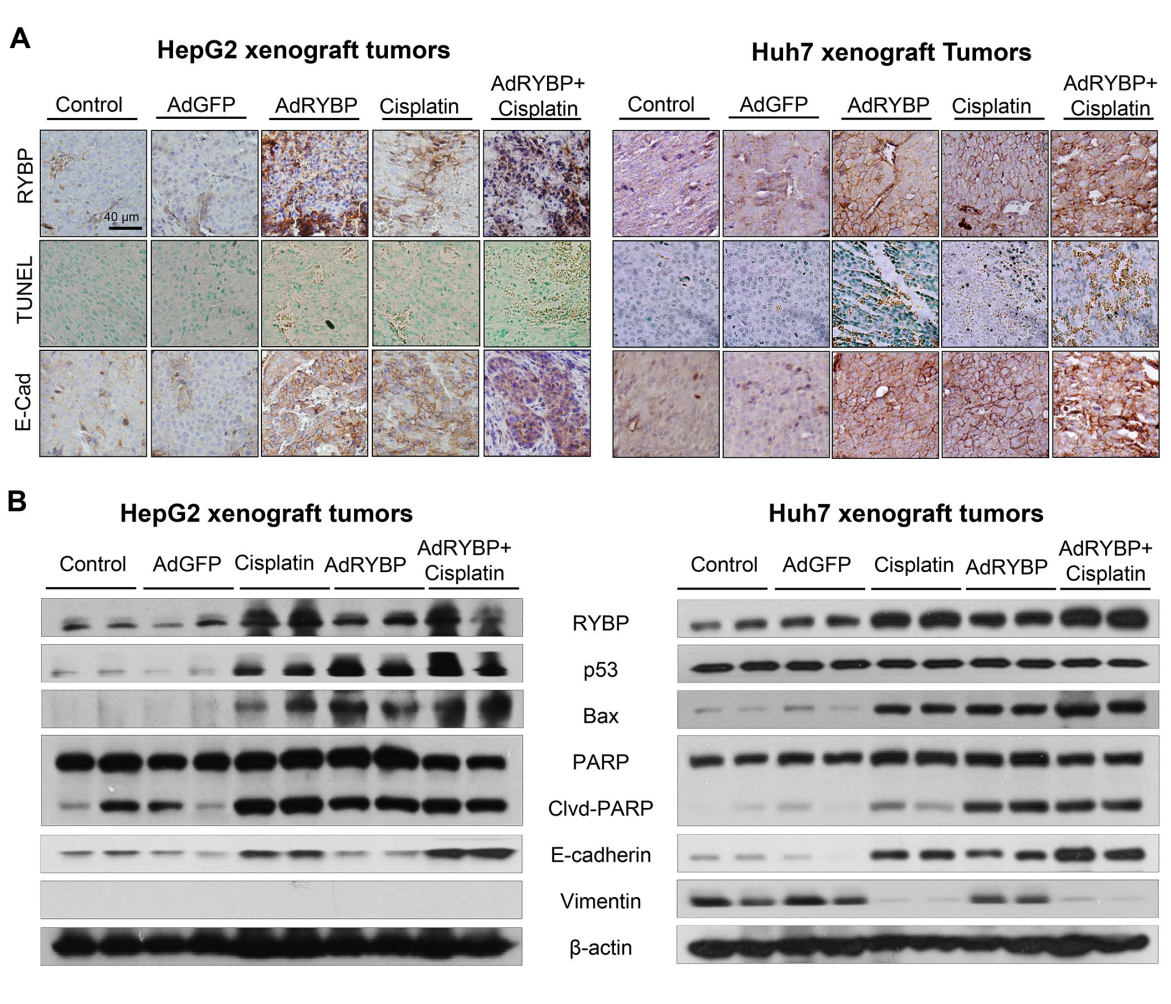
RYBP induces apoptosis *in vivo* A total of 2×10^9^ infectious particles (IP) of AdGFP or AdRYBP were administered by intratumoral injection every three days to nude mice bearing HepG2 (A) or Huh7 (B) tumors, and cisplatin was administered to the mice at 5 mg/kg by intraperitoneal injection once per week. At the end of the experiment, the tumors were excised, homogenized and further analyzed for the expression of proteins of interest by immunohistochemistry (A, all images represented the series sections) and Western blotting (B).

To investigate the mechanism(s) by which RYBP reduces tumor growth and sensitizes HCC tumors to cisplatin, we further evaluated the expression levels of the various apoptosis- and metastasis-related proteins *in vivo*. As shown in Fig. 7A, the results of the immunohistochemical staining showed that treatment with AdRYBP or cisplatin alone significantly induced RYBP and E-cadherin expression. Additionally, TUNEL staining demonstrated that there was an increase in apoptosis in the tumors of AdRYBP- and cisplatin-treated mice. When AdRYBP was used in combination with cisplatin, there were more TUNEL-positive cells than that in the tumors of mice treated with cisplatin alone. These observations were further confirmed by Western blotting (Fig. 7B). Consistent with the *in vitro* data, AdRYBP enhanced the cisplatin-induced expression of p53, Bax and PARP cleavage, suggesting that RYBP has an important role in determining the cellular response to cisplatin *in vivo*.

## DISCUSSION

In the present study, we have demonstrated a novel role for RYBP in the progression of HCC, and have elucidated the relationship between RYBP expression and the prognosis of HCC patients. We have made at least six novel discoveries: (1) RYBP is downregulated in HCC tissues and cell lines, and a low level of RYBP predicts a poor prognosis in HCC patients; (2) RYBP overexpression decreases the viability, inhibits colony formation, induces apoptosis, and inhibits the invasion of HCC cells; (3) apoptosis is the main mechanism by which the RYBP exerts its cytostatic effects in HCC cells; (4) AdRYBP infection decreases the growth of tumors in two different HCC xenograft models, and induces RYBP expression and modulates the levels of various other proteins, with changes similar to those observed *in vitro,* regardless of the p53 status of the tumor; (5) chemotherapeutic agents induce RYBP protein expression *in vitro* and *in vivo*; and (6) RYBP sensitizes HCC tumors to conventional chemotherapy through the induction of apoptosis both *in vitro* and *in vivo*. In light of the present evidence related to clinical cancer specimens and the pro-apoptotic role of RYBP, we concluded that RYBP may be a potential therapeutic target for HCC.

Integrative genomic profiling of human prostate cancer has revealed several regions of copy number loss, one of which spans the multigenic RYBP-containing region at 3p14 [19]. Another study revealed that the genetic loss of RYBP is associated with a poor outcome after chemoradiotherapy in human cervical cancer [20]. In contrast, RYBP was shown to be overexpressed in cases of primary classic Hodgkin's lymphoma and adult T-cell leukemia, indicating a possible tumor type-dependent expression and function of RYBP [21]. Of note, although the loss of the RYBP gene has been reported in several human cancers, these reports are very preliminary results in nature. Most of the studies are just a part of gene expression array and no mechanistic work has been done in any of those aforementioned studies [19,20]. In the present study, we used an extensive collection of HCC tumors to demonstrate that the RYBP expression was significantly downregulated in HCC tissues comparison with non-malignant peritumoral tissues. A low RYBP level was correlated with poor differentiation and an increased serum γGT level. HCC patients with low RYBP expression had poorer prognoses than patients with high RYBP expression. A multivariate analysis revealed that the RYBP expression was an independent and significant risk factor for survival. Of note, although the present series of HCC patients is well representative of a specific Chinese population (HBV infection), they may not be easily compared to HCC patients in other regions (e.g., USA, Europe, Japan and Africa) where most of the patients are infected with HCV and the vast majority of HCCs develop in a background of cirrhosis with multiple nodules. It is important that future studies validate our results with different cohorts of HCC patients with different clinical pathological features and from different regions, in order to ultimately determine the usefulness of RYBP as a biomarker and a potential therapeutic target for HCC worldwide. Additionally, since all the patients included in the present study were those undergoing surgery for removal of HCC tumors, whether the current results could be extrapolated to predict the clinical outcome of unresectable HCC cases needs further investigation. Currently, there are no randomized controlled trial (RCT) showing any benefits from systemic chemotherapies after resection in HCC. Thus, no uniformed, systemic chemotherapies were administrated to these HCC patients in the present study. Based on our *in vitro* and *in vivo* data from the present study, we believe that combination treatment with standard chemotherapeutic agents and RYBP targeted therapy may provide a new avenue to develop effective and safe management for patients with HCC.

RYBP exerts tumor-specific cell killing effects, but the underlying mechanism has not been fully investigated. RYBP has been suggested to be an inducer of apoptosis and a negative regulator of cell invasion [9, 13]. RYBP co-localizes with high performance parallel interface (Hippi) in a subset of neurons in the developing mouse brain, and may mediate or regulate the interaction between Hippi and caspase 8 [22]. RYBP also interacts with the viral apoptosis agonist Apoptin, and has been suggested to induce apoptosis preferentially in tumor cell lines, but not in normal fibroblasts or mesenchymal cells [11]. The pro-apoptotic functions of RYBP were further demonstrated by the fact that a high level of exogenous RYBP in Drosophila induces apoptosis by promoting the aggregation of the dFADD and DREDD (death-related ced-3/Nedd2-like) proteins, and activating the expression of the pro-apoptotic gene, reaper [10]. Similarly, mice homozygous null for RYBP die shortly post-implantation, and do not exhibit the normal apoptotic response accompanying implantation [23]. In this study, we demonstrated that overexpression of RYBP induces cell apoptosis and the expression of apoptosis-related proteins, while knockdown of RYBP attenuates this effect both *in vitro* and *in vivo*, suggesting that the essential role of RYBP in inhibiting cell growth may due to apoptosis. Our recent study demonstrated that RYBP binds to MDM2, prevents MDM2-mediated p53 degradation, and activates p53 transcriptional activity [16]. In p53 wide-type cells, RYBP selectively binds to MDM2 but not to p53, and the complex formed between the three proteins potentially acts as a reservoir of both p53 and MDM2. In response to DNA damage, this ternary complex possibly undergoes modifications, allowing release of p53 but not MDM2, thus reducing the rate of p53 degradation. The free p53 then mediates the transactivation of its downstream targets such as p21 and Bax, mediating cell cycle arrest and apoptosis. Thus, RYBP promotes apoptosis by regulating p53 activity in the cells with wild-type p53. In addition, RYBP binds to several apoptotic mediators and enhances apoptosis [9-12, 22]. In cells with non-functional p53, it is possible that RYBP also binds to Bax directly or it may have other targets. Further in-depth analyses of additional RYBP-interactive partners are needed to better understand the mechanism of action for RYBP's anticancer activities, especially in p53-independent manner.

In addition, the EMT plays a pivotal role in the dissemination of malignant hepatocytes and metastatic colonization [24]. The EMT is characterized by the loss of epithelial cell-cell adhesion proteins, such as E-cadherin, and the gain of mesenchymal markers, such as vimentin [25]. In this study, RYBP overexpression inhibited hepatoma cell invasion, and decreased the expression of E-cadherin and increased the expression of vimentin *in vitro* and *in vivo*. Our results suggest that the EMT might be a mechanism by which RYBP can induce HCC cell metastasis.

To date, almost all the available chemotherapeutic agents have been studied extensively in HCC. Cisplatin, 5-FU and doxorubicin are most extensively studied agents, showing the highest activity against HCC, but there is no substantial success and consistent result demonstrating that patients' overall survival is actually improved [26]. Since the monotherapy response is commonly limited, combination regimens have been studied in HCC. Among these, cisplatin-based regimens demonstrate a response rate of 28-45% and show a higher objective response rate than others [27]. Cisplatin's anticancer activity is associated with its DNA damaging effects and induction of cancer cell apoptosis [28, 29]. It has been reported that cisplatin-induced cell apoptosis is mediated by various signal transduction pathways, including death receptor signaling and mitochondrial signaling pathways [28, 29]. In the present study, we performed a preliminary evaluation of the therapeutic effects of RYBP by examining the effects of AdRYBP infection alone or in combination with cisplatin both *in vitro* and *in vivo*. Our results showed that cisplatin induced RYBP expression, and enforced expression of RYBP sensitizes HCC cells to cisplatin by inducing apoptosis both *in vitro* and *in vivo*. This combination regimen may provide an effective and safe therapeutic approach to HCC therapy. There have been a few previously published studies of the changes in RYBP expression in response to chemotherapeutic agents. For example, RYBP was shown to be upregulated by trichostatin A (TSA) and valproic acid (VPA) in v-FosFBR transformed 208F rat fibroblasts, a response mediated through the inhibition of histone deacetylation [13]. LAQ824, a small molecule inhibitor of histone deacetylases (HDACi), upregulated the RYBP expression in SKBr3 breast cancer cells through a miRNA27a-involving mechanism [15]. In our previous study, we showed that etoposide and doxorubicin induce RYBP expression in an osteosarcoma cell line (U2OS) [16]. In future studies, we will develop an optimized RYBP activator that might eventually make its way to clinical use following extensive testing in animals. We also plan to evaluate the potential of using RYBP as a potential biomarker for the diagnosis and prognosis of cancer, for assessing the progression of cancer, and for determining the response to therapy.

In conclusion, our results suggest that the downregulation of RYBP in HCC is a strong indicator of aggressiveness and a poor clinical outcome of tumors. Uncovering the novel functions of RYBP will shed light on the molecular mechanisms that regulate the growth and progression of HCC, and also provide a new avenue of research exploiting RYBP as a target for HCC therapy.

## MATERIALS AND METHODS

### Patients and Specimens

Archived tissue samples for tissue microarray (TMA) construction were obtained for a consecutive cohort of 400 patients who underwent surgery for curative resection of heptitis B virus (HBV)-related HCC in the Liver Cancer Institute, Zhongshan Hospital, Fudan University (Shanghai, China) between January 1, 2006 and January 1, 2007. The TMA study design was reviewed and approved by the Ethics Committee of Zhongshan Hospital, and informed consent was provided by each patient following the protocol approved by the Institutional Review Board [30]. None of the patients had signs of distant metastasis, and had not received any anticancer therapy before and after surgery. The follow-up procedures and postoperative treatments were performed according to a uniform guideline that was described previously [30]. The conventional clinicopathological variables are provided in Supporting Table 1. The tumor stage was determined according to the 2002 American Joint Committee on Cancer/Union for International Cancer Control (AJCC/UICC) tumor-node-metastasis (TNM) classification system. Tumor differentiation was graded by the Edmondson grading system. The liver function was classified based on the Child-Pugh scoring system. The data were censored at the last follow-up for patients without recurrence or death. The recurrence-free survival (RFS) and overall survival (OS) were defined as the interval between the time of surgery and that of recurrence or death, respectively. Fifty-two pairs of fresh frozen human primary HCC and matched adjacent non-cancerous liver tissue samples were obtained for a real-time quantitative PCR analysis of the RYBP expression.

### Cell Culture

Human HCC cell lines (HepG2, Hep3B, Huh7, SMMC-7721, MHCC97L [31, 32], MHCC97H [31, 32], MHCCLM3 [31, 32], and PLC/PRF/5), immortalized human normal hepatocytes (CL48), and human embryonic kidney (HEK293A) cells were maintained in Dulbecco's modified Eagle's media (DMEM), supplemented with 10% fetal bovine serum and 1% penicillin/streptomycin. The HepG2 and Hep3B cells were obtained from the American Type Culture Collection (Rockville, MD). The Huh7, SMMC-7721, and PLC/PRF/5 cell lines were kind gifts from Dr. Y. Yen (City of Hope, Duarte, CA). MHCC97-H, MHCC97-L, and MHCC-LM3 (with serial metastatic potential) cells were established as described previously [31, 32]. HEK293A cells were kind gift from Dr. W. Xu (Jiangsu University, Zhenjiang, China).

### Chemicals, Reagents, Antibodies, Plasmids, and siRNAs

All chemicals and solvents were of the highest analytical grade available. Cell culture supplies and media, fetal bovine serum (FBS), phosphate-buffered saline (PBS), and penicillin-streptomycin were obtained from Invitrogen (Carlsbad, CA). The anti-human p53, Bax, and poly (ADP-ribose) polymerase (PARP) antibodies were from Santa Cruz Biotechnology Inc. (Dallas, TX). The anti-human E-cadherin antibody was from BD Biosciences (San Jose, CA). The anti-human RYBP, vimentin, and β-actin antibodies were from Sigma (St. Louis, MO). Goat anti-mouse IgG (H+L) and goat anti-rabbit IgG (H+L) were obtained from Bio-Rad (Hercules, CA). The preparation of the Myc-RYBP expression vector was described previously. The RYBP siRNA pool and control siRNA pool were obtained from Dharmacon.

### Adenovirus Preparation, Purification, and Infection

AdRYBP, a replication-deficient adenovirus vector expressing RYBP, was generated as described [33]. The pAd-Easy1 adenovirus system was kindly provided by Dr. T-C He (University of Chicago). RYBP was subcloned into the pAd-track vector and then recombined with BJ5183/pAd-Easy1 competent cells by using the calcium transformation method [29]. The recombinants were identified and linearized with Pac I prior to transfection into HEK293A cells. Fourteen days after transfection, the viruses were collected and expanded for three cycles. The viruses were purified (Clontech, Mountain View, CA) and examined at different titers prior to further use. The MOIs (multiplicities of infection) were based on the titers determined for each cell line [33].

### Assays for *In Vitro* Anticancer Activity

Assays for cell viability (MTT assay) [34-36], colony formation [35, 36], apoptosis (Annexin V-FITC detection) [34-36], and cell invasion (transwell invasion assay) [36] were performed as described previously. In brief, 4-5×10^3^ cells per well were transfected with Myc-RYBP (3 and 5 μg), RYBP siRNA (20 and 50 nM), AdRYBP (300, 600, 900 and 1200 MOI), or their empty vectors for 72 h for MTT assay. For colony formation assay, cells were seeded in 6-well plates at 1×10^3^ cells per well, and were transfected with different plasmids for 24 h, then the cells were grown for another 10 days. To assess apoptosis using the apoptosis detection kit from BioVision (Mountain View, CA), 2-3×10^5^ cells were transfected with different plasmids and incubated for 48 h prior to analysis. Cells that were positive for Annexin V-FITC (early apoptosis) and PI (late apoptosis) were counted. To determine the effects of RYBP on the cell invasion, 2×10^4^ transfected cells (3 μg MycRYBP; 20 nM siRYBP, and 300 MOI AdRYBP) were seeded into the upper transwell chamber (BD Biosciences, CA) for 24 h, cells on the upper surface were removed and cells adhering to the lower membrane were stained with Mayer's Hematoxylin and Eosin solution and analyzed under a phase-contrast Olympus microscope (Olympus America Inc). The invading cells were counted in five different visual areas and the area of positive staining was measured using image analysis software (Image-Pro Plus 6.0, Media Cybernetics, Rockville, MD).

### Western Blotting and Real-Time Quantitative PCR

HCC cells and xenograft tumor tissue homogenates were collected and lysed in NP40 lysis buffer containing protease inhibitors (Sigma, St Louis, MO). Cell lysates were used for immunoblotting as described previously [34-36]. Total RNA was extracted using the Trizol reagent (Invitrogen, Grand Island, NY), and quantitative RT-PCR and Real-time PCR analysis were performed. The primer sequences used for the amplification of genes were as follows: RYBP sense: 5′-tttgcccagaaagacagctt-3′; RYBP antisense: 5′- gtcgtgcacatgccagtaac-3′; GAPDH sense: 5′-ggagtccactggcgtcttcac-3′ and GAPDH antisense: 5′-gaggcattgctgatgatcttgagg-3′ [16].

### Tissue Microarray, Immunohistochemistry and TUNEL Staining

Tissue microarrays were produced as described previously [30]. All HCC cases were histologically reviewed by HE staining, and representative tumor areas were premarked in the paraffin blocks, away from necrotic and hemorrhagic materials. Duplicate 1 mm diameter cylinders were included for each case. Sections of 4-μm thickness were taken on 3-aminopropyltriethoxysilane (APES)-coated slides. The immunohistochemical staining of serial TMAs was carried out as described previously [30]. Briefly, sections were dewaxed, hydrated, washed and incubated with primary and second antibodies. The reaction products were visualized with 3,3′-diaminobenzidine tetrahydrochloride and were counterstained with hematoxylin. The RYBP immunostaining intensities were scored as: 0, negative; 1, weak; 2, moderate; 3, strong. The negative grade represented no tumor cells showing positive immunostaining. For the analysis, the RYBP immunostaining intensities were classified as follows: the sections scored 0 and 1 were defined as the low expression group, and sections scored 2 and 3 were defined as the high expression group. The apoptosis of tumor tissues was detected using a TdT-mediated dUTP-biotin nick end labeling (TUNEL)-based *In Situ* Apoptosis detection kit (Trevigen, Inc, Gaithersburg, MD) according to the manufacturer's instructions [37].

### Development and Treatment of the HCC Xenograft Models

The animal study protocol was approved by the Institutional Animal Use and Care Committee (IACUC) of the Texas Tech University Health Sciences Center. Female athymic pathogen-free nude mice (nu/nu, 4-6 weeks) were purchased from Charles River Laboratories (Wilmington, MA). To establish HepG2 and Huh7 HCC xenografts, a total of 5 × 10^6^ cells (in 0.1 mL) were subcutaneously injected into the left inguinal area of the mice [34-36]. All animals were monitored for activity, physical condition, body weight, and tumor growth. When the tumor volume reached ~100 mm^3^, the mice bearing HepG2 and Huh7 xenografts were randomly divided into multiple treatment and control groups (10-15 mice/group). The *in vivo* treatment was planned based on up-to-date literature search and the real dose used in clinical treatment [26, 38, 39]. A total of 2×10^9^ infectious particles (IP) of AdGFP or AdRYBP were administered by intratumoral injection every three days; cisplatin (5 mg/kg) was administered by intraperitoneal (i.p.) injection weekly for about five weeks (HepG2) or 3 weeks (Huh7) [40-42]. The control group received the vehicle only. At the end of the experiments, the xenograft tumors, hearts, lungs, livers, kidneys, spleens and brains were removed, weighed, and snap-frozen for the Western blotting, immunohistochemistry, and TUNEL assays.

### Statistical Analysis

All preclinical data were analyzed using Prism software version 6 (Graph Pad software Inc., San Diego, CA, USA). The Student *t* test was used for comparisons between two groups. All clinical statistical analyses were performed with the SPSS 18.0 software program for Windows (IBM). Pearson's χ2 test and Fisher's exact test were applied to compare qualitative variables, and quantitative variables were analyzed by Student's t-test or Spearman's ρ rank correlation coefficient determination. A univariate analysis was performed using the Kaplan–Meier method (the log-rank test). A multivariate analysis was performed using a Cox multivariate proportional hazard regression model in a stepwise manner (backward, conditional). The model included all clinicopathological variables found to have significant prognostic value in the univariate analysis. A two-tailed value of p < 0.05 was considered statistically significant.

## SUPPLEMENTARY MATERIAL FIGURES AND TABLES


