# Supplementary material for: RYBP expression is associated with better survival of patients with hepatocellular carcinoma (HCC) and responsiveness to chemotherapy of HCC cells in vitro and in vivo
Source: Oncotarget. 2014 Oct 18;5(22):11604–19. doi: 10.18632/oncotarget.2598 (PMC4294343; doi:10.18632/oncotarget.2598)
Supplement: Supplementary file 1 [file oncotarget-05-11604-s001.pdf]

RYBP expression is associated with better survival of patients with hepatocellular carcinoma (HCC) and responsiveness to chemotherapy of HCC cells *in vitro* and *in vivo*

Supplementary Material

Supplementary Table 1: The clinicopathological features of the HCC patients

Variables	Results
Age, years (median, range)	53 (18-78)
Gender (male/female)	334/66
Virus infection (HBV/(-)/unknown)	356/41/3
Liver cirrhosis (no/yes)	62/338
Preoperative AFP, ng/ml (median, range)	94.5 (0.8-60500.0)
Preoperative γGT (U/L)	52.0 (10.0-812.0)
Tumor multiplicity (single/multiple)	340/60
Tumor size, cm (median, range)	4.0 (0.4-22.0)
Child-Pugh classification (A/B)	379/21
Vascular invasion (absent/present)	271/129
Tumor encapsulation (complete/incomplete)	220/180
Tumor differentiation (well/poor)	290/110
AJCC/UICC TNM stage (I/II/III)	237/125/38
BCLC stage (A/B/C)	151/120/129

Abbreviations: HBV, hepatitis virus B; AFP, alpha-fetoprotein; γGT, gamma-glutamyl transferase; AJCC/UICC, American Joint Committee on Cancer/Union for International Cancer Control; TNM, tumor-node-metastasis; BCLC, barcelona-clinic liver cancer

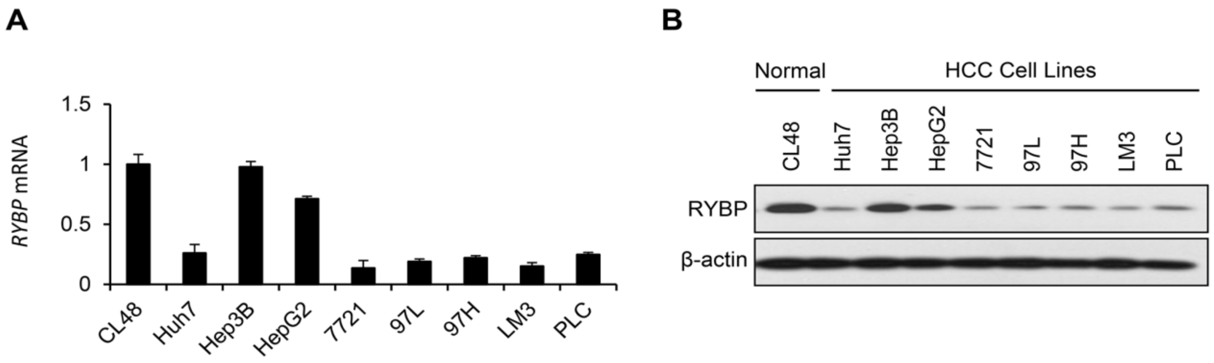

Supplementary Fig. S1: The expression of RYBP in HCC cell lines. (A). Quantitative real-time analyses of the RYBP mRNA levels in non-malignant human hepatocytes (CL48) and hepatoma cell lines. (B). Immunoblotting assays of the RYBP protein expression in human normal hepatocytes and hepatoma cell lines.

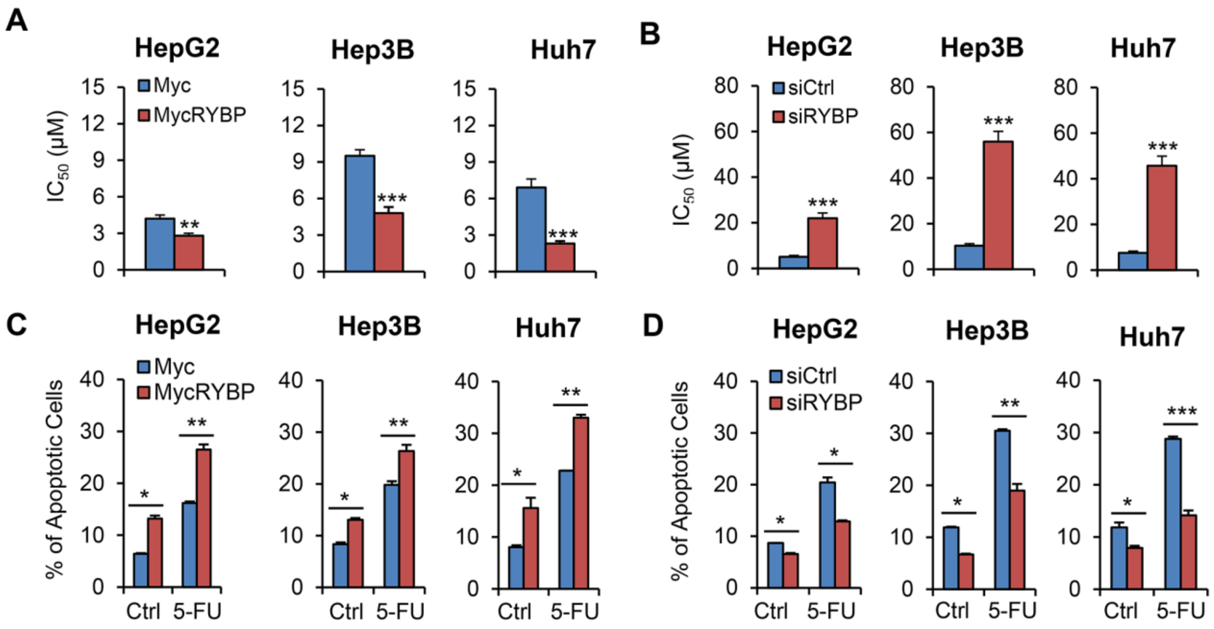

Supplementary Fig. S2: RYBP overexpression and knockdown affect the chemosensitivity of HCC cells to 5-FU. HepG2, Hep3B and Huh7 cells were treated with 5-FU and the RYBP plasmid (24 h) or with RYBP siRNA (48 h), then the IC_{50} values was determined by the MTT assay (A and B) and cell apoptosis was determined by the Annexin V-FITC method (C and D).

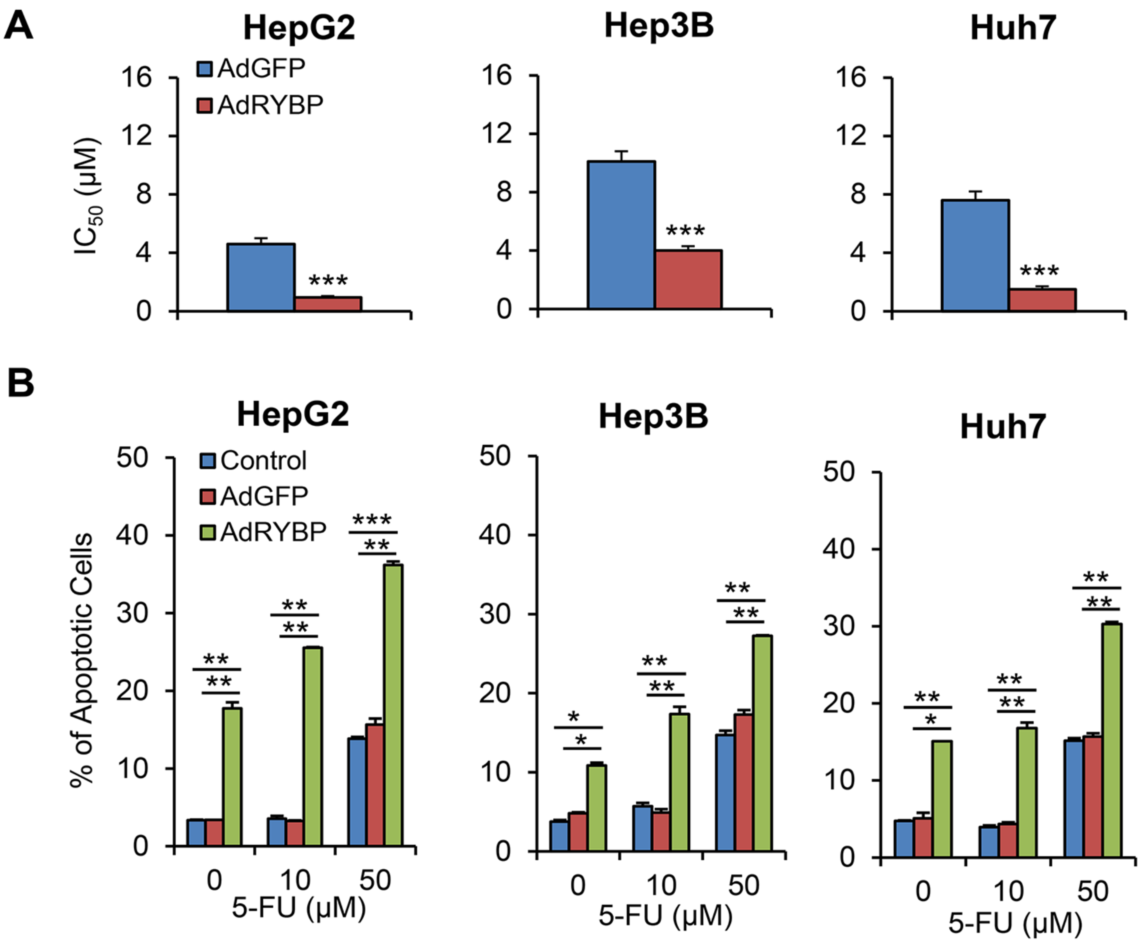

Supplementary Fig. S3: AdRYBP infection increased the chemosensitivity of HCC cells to 5-FU. HepG2, Hep3B and Huh7 cells were treated with 5-FU and the AdRYBP plasmid (24 h), then the IC_{50} values were determined by the MTT assay (A) and cell apoptosis was determined by the Annexin V-FITC method (B).
